# The secretor status of blood group antigens in the saliva in people with oral cancers: a systematic review

**DOI:** 10.1186/s13643-023-02399-8

**Published:** 2024-01-02

**Authors:** T. Walpola, K. L. T. D. Jayawardene, I. Weerasekara

**Affiliations:** 1 Department of Medical Laboratory Science, Faculty of Allied Health Sciences, University of Peradeniya, Peradeniya, Sri Lanka; 2 Faculty of Health and Social Sciences, Western Norway University of Applied Sciences, Bergen, Norway; 3 School of Health Sciences, The University of Newcastle, Callaghan, NSW Australia; 4 School of Allied Health Science and Practice, Faculty of Health and Medical Sciences, The University of Adelaide, Adelaide, SA Australia

**Keywords:** Secretor status, Oral cancer, ABO blood group, Saliva

## Abstract

**Background:**

Human ABO blood group type and the antigenic secretor status are hypothesized to associate with oral diseases including oral cancer. Secretor status is the ability of individuals to secrete blood group antigens into body fluids. This study aimed to evaluate the secretor status of ABO antigens of saliva in patients with oral cancers or oral potentially malignant disorders (OPMDs) relative to healthy adults.

**Methods:**

A systematic and comprehensive online search from inception to April 28, 2022, was carried out in MEDLINE, Embase, PsycInfo, and Emcare. The language was limited to English. Yielded records were screened by two independent reviewers at the title and abstract phase and at full-text screening. Studies investigating adults (≥ 18 years) with oral cancers or oral potentially malignant disorders compared to adults free of oral cancer were included in this study. Data were extracted according to the planned objectives. Methodological quality was assessed, and the findings were analyzed narratively. Meta-analyses were conducted to pool the odds of the non-secretor status of oral cancers and OPMDs compared to healthy adults.

**Results:**

The search included a total of 34 studies from three databases. Nine duplicates were removed. During the title and abstract screening, 11 irrelevant studies were excluded. Twelve studies were screened during the full-text screening, and eight articles were eligible to be included in the final analysis. A pooled odds ratio (OR) of 3.80 (95%CI, 1.53–9.44) was estimated when pooled 1254 oral cancers and oral potentially malignant disorders patients compared to 666 healthy adults.

**Discussion and conclusion:**

The odds of being a non-secretor appear to be approximately 3.8 times higher in patients with oral cancers and oral potentially malignant disorders compared to healthy adults. The lack of ABO blood group antigens in body fluids of non-secretors is more exposed to exogenous antigens than secretors. The host-parasite interactions of secretors and non-secretors underlying oral cancer and other diseases may be evidence to support or refuse them. Clinicians may use the secretor status as a detection test during their regular oral check-ups for high-risk populations for oral cancers. Non-secretors can be given more attention considering them as high-risk groups, and in terms of prognosis, differences between these two groups may be expected.

**Supplementary Information:**

The online version contains supplementary material available at 10.1186/s13643-023-02399-8.

## Background

There are around 33 blood group systems that are being discovered and listed by the international society of blood transfusion [1]. ABO and Rh blood group systems are considered as the clinically most significant out of all the systems [2]. Blood group is a genetically determined factor, and it is a unique character of every individual [2] that remains constant throughout one’s life [3].

ABO blood group system consists of antigens A, B, and H which are usually present on the red blood cell membrane [2]. Expression of ABO antigens is controlled by 3 separate genetic loci; the ABO gene on the long arm of chromosome 9, FUT 1 (H), and FUT 2 (Se) genes on chromosome 19. Each gene is responsible for coding a particular enzyme (glycosyl transferase) which attaches to its specific monosaccharide precursor [4].

The ability to secrete A, B, and H substances in water-soluble form to the body fluids is controlled by FUT 2 (Se) gene. The individuals who possess this genetic ability are called secretors, while those who do are not called non-secretors. Generally, in a population, there are around 80% secretors and 20% non-secretors. In secreters, blood group antigens are secreted into saliva and other body fluids like sweat, digestive secretions, breast milk, and tears [5]. The secretor status of a person is controlled by a pair of allelomorphic genes Se and se. Se is dominant over se [6]. Secretor status is not dependent on the blood group [7]. Secretor status can be determined by detecting the presence of the H antigen in saliva. There are no H antigens present in the saliva of non-secretors [8].

Previous studies have investigated the existence of allele-level associations with human secretory state independent of the gene of interest in the present review. They have looked into alleles’ effects on the secretor status of humans in addition to the Se/se (FUT 2) gene. A, AB, and B blood types have been linked to an increased risk of pancreatic cancer, according to one study [1]. Furthermore, a gene-dose effect has been identified, wherein each additional A or B allele is linked to a higher risk than the O allele. In order to further explore a potential function for ABO glycosyltransferase and ABO antigen expression in pancreatic cancer etiology, researchers have looked at variations in the ABO and FUT2 genes. These were tested with matched controls in terms of year of birth (±5 years), gender, self-reported race and ethnicity, and DNA source (peripheral blood or buccal cells).

Secretor status was detected in different studies in people with different pathological conditions including chronic atrophic oral candidosis among diabetes mellitus patients [9], peptic ulcers, vaginal candidosis, oral changes like oral submucous fibrosis (OSF), dental caries, periodontal diseases, and certain autoimmune diseases like Pemphigus vulgaris [10]. In addition to these, we found several studies supporting the use of detection of oral cancers by secretor status [11, 12]. Oral cancer is a global major health problem, especially in developing countries. Worldwide, it is ranked at the top among the most common cancers in human beings [13]. In South East Asia, more than 10^5^ new cases of oral cancer are reported annually accounting for about 40% of all cancers compared to the 2–5% reported in Western countries [6].

There are several host risk factors affecting the development of oral cancers such as alcohol consumption, tobacco smoking, nutritional status, viral infections such as human papilloma virus (HPV), and ABO antigens [14]. Oral cancer appears as a growth or sore in the mouth including the surface of the tongue, the inside of the cheeks, the roof of the mouth (palate), and lips or gums. However, secretor status is still a hypothesis which has to be proven as a risk factor [6]. There were a number of studies that reported a relationship between the ABO blood group type and the risk of getting oral cancers [11, 12] with inconsistent conclusions. However, no systematic review is available. Therefore, the present review aims to systematically assess the ABO blood group secretor status in people affected by oral cancers or oral potentially malignant disorders. The hypotheses for this systematic review are null hypothesis (H_0_), oral cancers or oral potentially malignant disorders are not affected by the ABO blood group secretor status of people, and alternative hypothesis (H_1_), oral cancers or oral potentially malignant disorders are affected by the ABO blood group secretor status of people.

## Methods 

A systematic and comprehensive search on MEDLINE, Embase, PsycInfo, and Emcare was carried out for the published literature from inception to April 28, 2022. The search strategy for MEDLINE (Appendix 1) was built up based on the key terms related to ‘secretor status’, ‘oral cancer’, ‘secretor status’, ‘saliva’, and ‘ABO blood group antigens’. This was adapted to other databases in OVID. The language was restricted to English. The data search was exported to COVIDENCE systematic manager software, and the duplicates were removed. The review was registered in the International Prospective Register of Systematic Reviews (PROSPERO) on February 15, 2022 (CRD42022322642).

Studies were included if (1) a clear extractable typing of ABO blood type is identified, (2) studies contain the assessment of secretion of ABO blood group antigens in the saliva of adults (≥ 18 years) with oral cancers, and (3) have a comparison group with adults free of oral cancer. Any study assessing people with no oral cancer, or any other dental disorder, or below 18 years of age was excluded. Based on this eligibility criteria, two independent researchers screened the titles and abstracts first and then the full texts. Any discrepancies were resolved by consensus in both stages.

Data were extracted from each eligible study to a pre-defined criteria in an Excel sheet. Data extracted based on the publication details, study design and setting, sample size, population characteristics, intervention characteristics (details about the saliva antigen test), outcomes (e.g., incidence, risks, and prevalence), and future research suggestions by the first author.

**Fig. 1:**
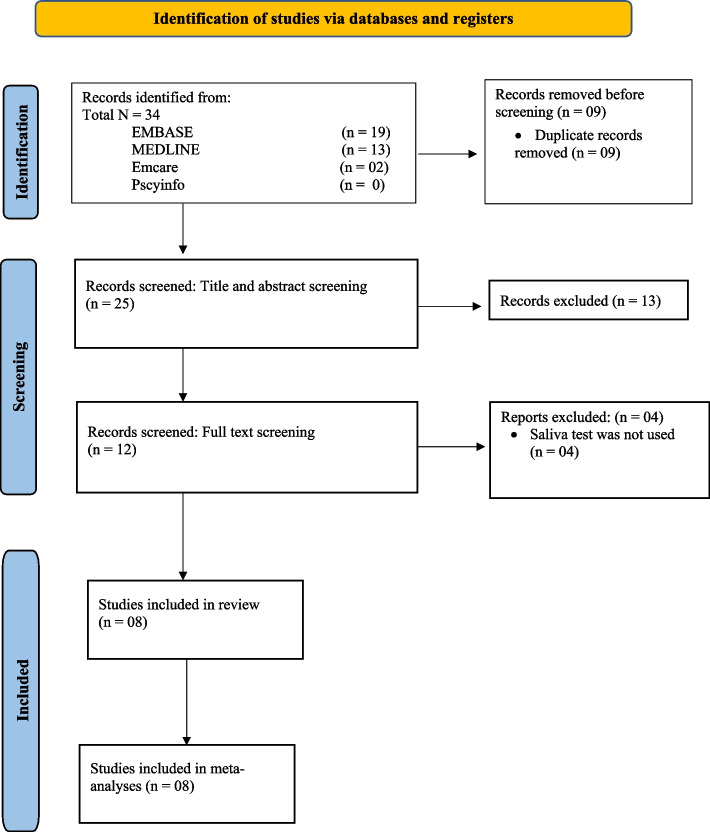
PRISMA flowchart [15] (n = Number of studies)

**Fig. 2:**
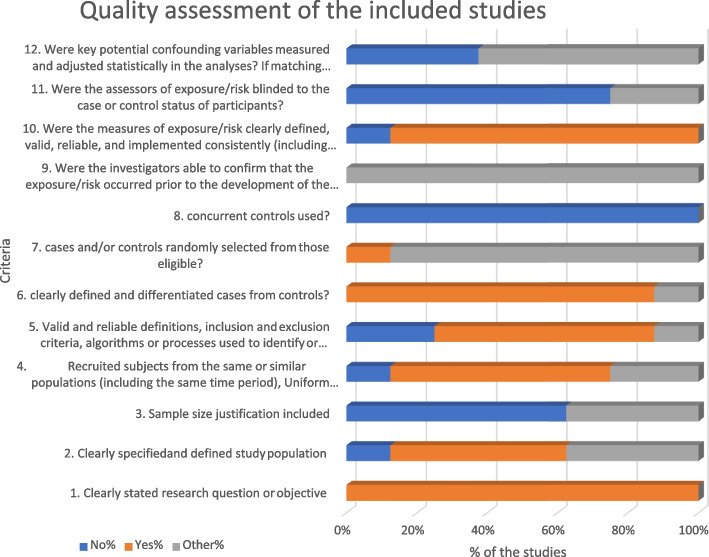
Quality assessment of the included studies

**Fig. 3:**
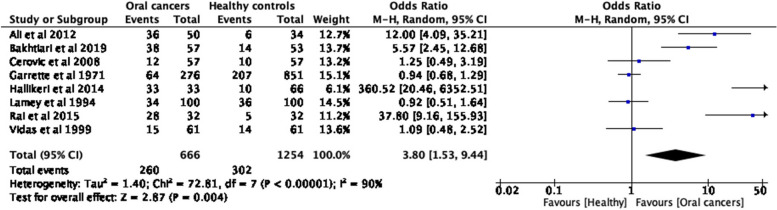
Odd ratios (95% CI) of non-secretor status in oral cancers and premalignant oral conditions compared to healthy controls, by pooling data from 8 studies (*n* = 1920). *Note*: In the study of Hallkeri et al., 2014, data of two groups; ´patients with a tobacco-related habit, but no lesions´ and ´healthy controls´ were combined as the control group because there were no pathological changes in both the groups

**Fig. 4:**
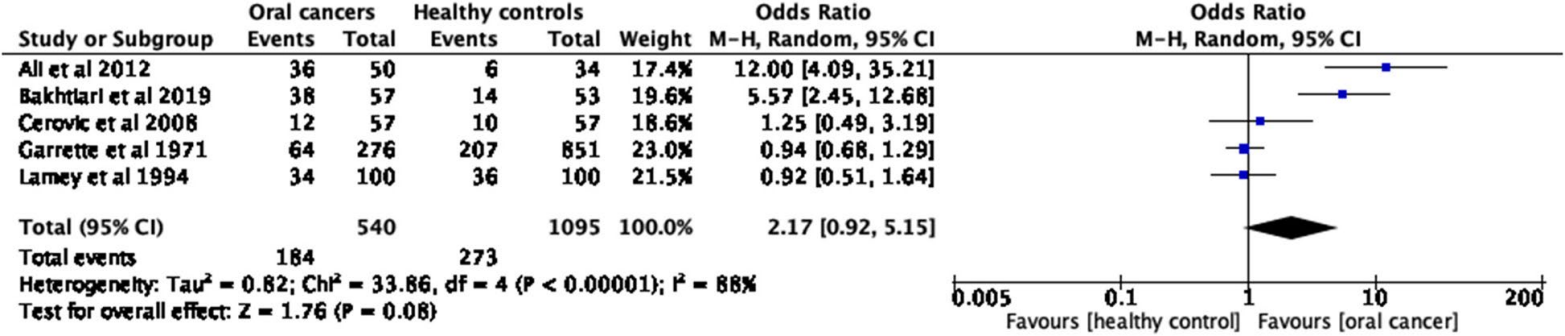
Odd ratios (95% CI) of non-secretor status in oral cancers compared to healthy controls, by pooling data from 5 studies (*n* = 1635)

**Fig. 5:**
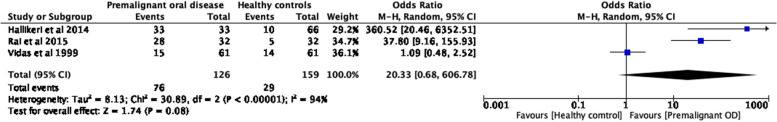
Odd ratios (95% CI) of non-secretor status in premalignant oral conditions compared to healthy controls, by pooling data from 3 studies (*n* = 285)

A narrative analysis was carried out summarizing the evidence on secretors of ABO blood group antigens in the saliva in people with oral cancers, compared to non-secretors. The summary was tabulated as per the research objectives. Further, we have conducted three separate meta-analyses (Figs. 1, 2, 3, 4 and 5) to show the odds of being a non-secretor in oral cancer and/or pre-malignant oral diseases. The Mantel–Haenszel (M-H) meta-analysis was performed with the random-effects model because of the observed substantial heterogeneity. The *I*^2^ values were used to assess the heterogeneity, and a value greater than 75% was considered substantial heterogeneity (https://handbook-5-1.cochrane.org/whnjs.htm).

The quality of studies was assessed using Quality Assessment Tools of Systematic Reviews and Meta-Analyses developed by the National Heart, Lung, and Blood Institute (NHLBI) in 2013 (https://www.nhlbi.nih.gov/health-topics/study-quality-assessment-tools). All included studies were rated based on a few main quality assessment tools in the form of 13 questions, including research question, study population, target population and case representation, sample size justification, groups recruited from the same population, inclusion and exclusion criteria, case and control definition, random selection of study participants, concurrent controls, exposure assessed prior to outcome measurement, exposure measures and assessment, blinding of exposure assessors, and statistical analysis. The risk of potential for selection bias, information bias, measurement bias, or confounding was considered during the appraising process. Two researchers independently assessed the reviews’ quality using the NHLBI quality assessment tool. Adherence to each item was rated as follows: yes, no, not reported, cannot determine, and not applicable (such as when a meta-analysis was not conducted). The overall confidence in the results of the review is rated as ‘good’, ‘fair’, or ‘poor’. High risk of bias reflects a methodology with a poor quality while a low risk of bias reflects a good methodological quality.

## Results

The search resulted in a total of 34 studies from 4 databases: MEDLINE (*n*= 13), Embase (*n*= 19), and Emcare (*n*= 2). There were no relevant studies were found in Pscyinfo. Nine studies were subsequently removed because they were duplicates. During the title and abstract screening, 11 irrelevant studies were excluded. During the full-text screening, 12 studies were screened and a total of eight articles were eligible to be included in the final analysis. Four studies were excluded because the saliva test was not used in those studies and instead, Histo-antigens were tested [16–19]. PRISMA flow chart shows the study flow (Fig. 1). The details of the methodological quality and classification of the studies are shown in Fig. 2.

The characteristics of the included studies are shown in Table 1. The studies were conducted in Ireland, Iran, Iraq, India, and Croatia and published between 1994 and 2019. The included eight studies were assessed under 13 quality assessment criteria and were categorized as Good, Fair, and Poor studies in quality. Five studies were considered as with a good quality [11, 12, 20–22], while the other 3 were considered as with a fair quality [6, 23, 24]. Oral cancer studies were not only restricted to the buccal cavity, but ranged from head and neck cancers to cancers in the buccal cavity, salivary glands, palate, tongue, and pharynx. The experimental group of the Iranian study had patients diagnosed with head and neck cancer while the staff members of the same hospital were considered as the controls [21]. In the study conducted in Iraq, patients diagnosed with oral cancers included the buccal cavity, tongue, salivary glands, and oropharynx and were compared with apparently healthy volunteers with no oral lesions [11].

**Table 1:** Characteristics of included studies

Author/Year Study title	Design	Country	Age		Sample size			Gender (Male/ Female)	
			Cases	Control	Total	Cases	Control	Cases	Control
Ad'hiah et al. 2018 [11] Secretor status of ABH blood group Ag s in a sample of Iraqui cancer patients	Case-control study	Iraq	53.9±13.1	44.2±12.7	84	50	34	23/27	17/17
Bakhtiari et al. 2019 [21] Salivary secretes status of blood group Ag s in patients with head and neck cancer	Case-control study	Iran	58.69±14.8	49.13 ±11.1	110	57	53	32/25	27/26
^c^Cerović et al. 2008 [12] Examining the presence of ABO (H) Ag s of blood types in saliva of patients with oral cancer	Case- control study	Croatia	62	60	114	57	57	47/10	47/10
Garrett et al. 1971 [25] Blood groups and secretes status in patients with salivary gland tumors	Case- control study	UK	NR	NR	1961	407	1554	NR	NR
^a^Hallikeri et al. 2014 [20]	Case- control study	India	NR	NR	99	33	66	NR	NR
Analysis of salivary secretor status in patient with oral submucous- fibrosis									
Lamey et al. 1994 [6] Secretor status and oral cancer	Prospective	Ireland (Sri Lankan population)	NR	NR	200	100	100	80/20	80/20
^b^Rai et al. 2015 [23] Assessment of ABO blood grouping and secretor status in the saliva of the patients with OPMD s	Cross- sectional study	India	NR	NR	64	32	32	NR	NR
^c^Vidas et al. 1999 [24] Examining the secretor status in saliva of patient with oral pre- cancerous lesions	Cross- sectional study	Croatia	61	61	122	61	61	NR	NR

*Abbreviations*: *NR* Not Reported, *OPMD* Oral Potentially Malignant Diseases

^a^In this study, data of two groups; ´patients with a tobacco-related habit, but no lesions´ and ´healthy controls´ were combined as the control group because there were no pathological changes in both the groups

^b^This study reported 45 in each group in the abstract, and 32 in the results section. We recorded the numbers in the results section

^c^These studies have reported only the mean values of age

Our interested outcome is the secretor status of the ABO blood group antigens in the saliva in people with oral cancers or oral potentially malignant conditions. The secretor status is described as the ability of individuals to secrete blood group antigens into body fluids. We found that out of 8 studies, 4 studies reported a higher percentage of non-secretors presented with oral cancers or oral potentially malignant [2–5] conditions while others reported the opposite. Individual results of the presence of the secretor status of ABO blood antigens and oral cancer or oral potentially malignant disorders were recorded in Table 2. Almost all the studies have shown that there was a high percentage of non-secretors in the case group when compared to the control group, thereby showing that non-secretors have a higher chance of developing oral cancer. The studies that have assessed the relationship between secretor status and gender in developing oral cancer have found that more males in the study group are secretors when compared to female secretors in the study group.

**Table 2:** The secretor status and ABO blood type of the oral cancers compared to a healthy control group

Study	Secretor status n (%)				Male %				Female %				ABO blood type %							
	Oral cancers/Pre-malignant oral diseases		Healthy controls		Secretors		Non-secretors		Secretors		Non-secretors		Cases				Controls			
	Secretors	Non-secretors	Secretors	Non-secretors	Cases	Control	Cases	Control	Cases	Control	Case	Control	A	B	A B	O	A	B	A B	O
Ad'hiah et al. 2018 [11]	14 (28)	36 (72)	28 (82)	06 (17)	NR	NR	NR	NR	NR	NR	NR	NR	N R	N R	N R	N R	N R	N R	N R	N R
Bakhtiari et al. 2019 [21]	19 (33)	38 (67)	39 (74)	14 (26)	NR	NR	NR	NR	NR	NR	NR	NR	N R	N R	N R	N R	N R	N R	N R	N R
Cerović et al. 2008 [12]	45 (79)	12 (21)	47 (83)	10 (18)	83	85	17	15	60	70	40	30	N R	N R	N R	N R	N R	N R	N R	N R
^b^Garrett et al. 1971 [25]	212 (77)	64 (23)	644 (76)	207 (24)	NR	NR	NR	NR	NR	NR	NR	NR	42	10^b^		4 8	4 1	11^b^		48
^a^Hallikeri et al. 2014 [20]	00 (0)	33 (100)	56 (85)	10 (15)	NR	NR	NR	NR	NR	NR	NR	NR	36	27	3	3 3	5 1	30	12	6
Lamey et al. 1994 [6]	66 (66)	34 (34)	64 (64)	36 (36)	NR	NR	NR	NR	NR	NR	NR	NR	23	29	6	4 2	1 9	20	2	59
Rai et al. 2015 [23]	28 (87)	04 (13)	27 (84)	05 (16)	13	34	75	13	0	50	13	3	42	20	9	2 9	3 8	29	4	29
Vidas et al. 1999 [24]	46 (75)	15 (25)	47 (77)	14 (23)	NR	NR	NR	NR	NR	NR	NR	NR	N R	N R	N R	N R	N R	N R	N R	N R

*Abbreviations*: *NR* not reported

^a^In this study, data of two groups; ´patients with a tobacco-related habit, but no lesions´ and ´healthy controls´ were combined as the control group because there were no pathological changes in both the groups

^b^This study has reported data as a percentage separately for group A, O, and ´AB+B´ together

Our first meta-analysis shows that the odds of being a non-secretor is 3.8 (95% CI, 1.53–9.44) times higher in patients with oral cancers and oral potentially malignant disorders compared to healthy adults (*p* = 0.004) (Fig. 3). Two other meta-analyses were conducted to see the same measure separately for oral cancers (*p* = 0.08, Fig. 4) and for oral potentially malignant disorders (*p* = 0.08, Fig. 5). However, none of the meta-analyses was found to be statistically significant.

Regarding the findings of the quality assessment, almost all the studies have clearly stated their research question or objective and concurrent controls were not used by almost all the studies. Around 85% of the studies have clearly defined and differentiated cases from the controls. However, 75% of the studies has not stated whether the measure of exposure/risk is clearly defined, valid, reliable, and implemented consistently across all study participants.

**Fig. 6:**
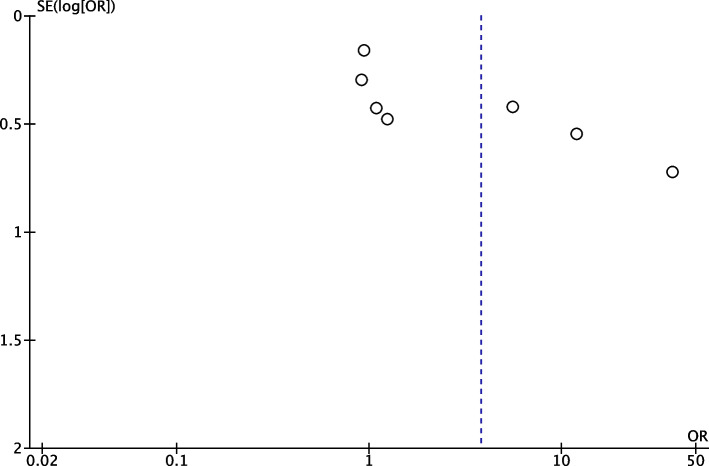
Funnel plot for the included studies in meta-analyses

There is a visual asymmetry in the funnel plot (Fig. 6), indicating potential publication bias, or other factors like heterogeneity between studies, or differences in study quality.

## Discussion

We aimed to evaluate the secretor status of the ABO blood group antigen in oral cancer and oral potentially malignant disorders in comparison to healthy adults. Half of the included studies in the review indicated that there seem to be higher odds of non-secretor status in oral cancers and oral potentially malignant disorders. Our meta-analysis also confirmed the statistical significance of this. These findings are clinically relevant in many different ways. There is a potential for this test to be used as a diagnostic marker or risk factor. Clinicians may consider screening for ABO blood group secretor status in people at risk to aid in early detection of oral cancers. Also, individuals with non-secretor status may be at increased risk and therefore can be used in risk assessments which may help in making better clinical decisions during the screening, surveillance, and prevention strategies.

There are several hypotheses were proposed reasoning the relationship between secretor status and underlying risk of oral cancers. It has been found that due to the lack of ABO blood group antigens in body fluids of non-secretors, they are more exposed to endogenous and exogenous antigens than secretor people [10]. Non-secretors are more prone to peptic ulcers, vaginal candidosis, oral pre-malignant lesions like OSF (oral submucuous fibrosis), dental caries, periodontal disease, and certain autoimmune diseases like Pemphigus vulgaris, the most common type of life-threatening autoimmune disease [10]. Secreted blood group antigens in body fluids are reported to have several pathogen evasion mechanisms including reduction of pathogen attachment due to the structure of ABO (H) antigen thereby reducing the infectivity [8].

Several other hypotheses have also been proposed to explain the host-parasite interactions of secretors and non-secretors underlying oral cancer and other diseases and evidence to support or refute them [26]. Some such hypotheses are that anti-A and anti-B isohemagglutinins are ‘natural’ bactericidal or opsonic antibodies for some strains of microorganisms, the ABO or Lewis antigens in the body fluids of secretors are receptors for adhesins on the surface of some strains of microorganisms and they interfere with the attachment to target cells, some products of the secretor gene alter the receptor for some microbial adhesins or molecules near these receptors reducing the numbers of microorganisms bound, the lower levels of serum and salivary IgA reported for non-secretors contribute to a compromised state at their mucosal surfaces, and the secretor gene and the structural gene for the third component of complement (C3) are in the same linkage group—there might be differences in the levels of C3 found in secretors and non-secretors [26].

Additionally, actual biological consequences have been suggested previously in humans for the reduced enzymatic activity of the A_2_ glycosyltransferase or A _1_ glycosyltransferase, most clearly in relation to circulating levels of von Willebrand factor (vWF) and the risk of venous thromboembolism (VTE). The ABO glycosyltransferase attaches sugar residues to vWF, which is subsequently secreted into the circulation. In subjects with blood group O, vWF is cleared more quickly, resulting in approximately 25% lower levels of circulating vWF than in subjects with blood groups A or B. Therefore, the differing glycosyltransferase activity resulting from the *A* alleles is biologically relevant to a disease outcome (i.e., VTE) and seems to be directly related to the efficiency of glycosyl group transfer to a target molecule (i.e., vWF) which we believe further implicates ABO glycosyltransferase activity even in the pathogenesis of oral cancer.

Previously, it was suggested that differences in the activity of certain enzymes, A2 glycosyltransferase or A1 glycosyltransferase, could have effects on human health. This is most evident in how these enzymes impact the levels of von Willebrand factor (vWF) in the bloodstream and the risk of venous thromboembolism (VTE).

The ABO glycosyltransferase is responsible for adding sugar molecules to vWF, which is then released into the bloodstream. In individuals with blood type O, vWF is removed from the bloodstream more quickly, leading to around 25% lower levels of vWF compared to those with blood types A or B. So, the different enzyme activities resulting from the A alleles are biologically significant in determining the risk of VTE, and this is linked to how efficiently sugar groups are added to a specific molecule, vWF. This suggests that ABO glycosyltransferase activity may even play a role in the development of oral cancer.

We found another similar study conducted on the ABO blood group and different cancers including gastric cancer, esophageal cancer, breast cancer, ovarian cancer, nasopharyngeal cancer, and pancreatic cancer [27]. That study has concluded that blood group A is associated with an increased risk of cancer, and blood group O is associated with a decreased risk of cancer [27], while the present study has not shown such a specific relationship. Further, another meta-analysis suggested that blood group A has a greater risk for developing oral cancer and oral potentially malignant disorders [28], while blood group O was associated with lower chances of oral cancer. No association was observed between the ABO blood group system with oral submucous fibrosis [28]. Further, there is a discrepancy among published observations regarding the effect of the ABO blood group on periodontium health [29]. It also stressed further studies with a large sample population to build more robust evidence, which remains the same conclusion of our review.

As described in the introduction, some studies have paid attention to the genetic basis of secretor status at the allele level to reveal a relationship between secretor status with diseases. Blood group A has 2 different alleles A1 and A2. First off, the A1 glycosyltransferase, which has higher enzymatic activity than the A2 glycosyltransferase, appears to be primarily responsible for the relationship of cancer risk with the A allele [1]. In a study conducted on secretor status and pancreatic cancer, they have identified that although producing blood group A, the less active A2 glycosyltransferase did not raise the risk of pancreatic cancer when compared to the inactive O glycosyltransferase [1].

Recent epidemiological studies and the PanScan GWAS revealed that persons with blood groups other than O (A, AB, and B) have a higher chance of developing pancreatic cancer than those with blood group O. It is noteworthy that the majority of statistically significant single-nucleotide polymorphisms (SNPs) found in the PanScan GWAS were situated in the ABO gene’s first intron, in close proximity to the single base loss that determines blood group O. A follow-up analysis discovered a gene-dose effect, whereby each extra non-O allele doubled a subject’s risk for pancreatic cancer, and a strikingly consistent rise in risk for non-O/O genotypes across 12 distinct prospective cohorts. All of these demonstrate the existence of allele-level associations with the human secretory state independent of the gene of interest in the present study.

In terms of discussing the quality assessment of the included studies, most of the criteria like pre-defined and specified eligibility criteria, use of comprehensive, systematic literature search strategy, studies included with important characteristics, and results of each study were well followed. The common methodological drawbacks were omission of prospective protocol submission or publication, use of inappropriate search strategy, lack of independent and dual literature screening and data extraction (or unclear methodology), and lack of reasons for study exclusion (or rationale unclear). These should be addressed in future studies.

The current review has a limitation to acknowledge. The search for the language has been restricted to English; therefore, we may have missed other important literature published in other languages. However, the value of this systematic review is noteworthy in terms of its comprehensive search and quantitative analysis. Therefore, it is obvious that there may be other factors such as alleles associated with oral cancers as we have discussed previously in the discussion and introduction section; however, our review objective was to find out any association of oral cancers with the phenotypical secretor status.

In conclusion, our findings can be used by cancer screening programs to facilitate the early identification of potential high-risk groups of cancer in an easier and a non-expensive way. Health care providers in cancer screening and preventive programs should take these findings into careful consideration. Clinicians may use secretor status detection tests during their regular oral check-ups for high-risk populations for oral cancers. Non-secretors can be given more attention considering them as high-risk groups, and in terms of prognosis, differences between these two groups may be expected. Further studies with increased number of samples with some consideration of other confounding factors such as alcohol drinking and tobacco smoking are warranted to explore the underlying mechanism for these findings, prognostics, and detection of the relationship between oral cancer with ABO blood group type and their subgroups. Potential publication bias is indicated by the funnel plots, and therefore the suggestions should be treated with caution.

### Supplementary Information

**Additional file 1. **

## Data Availability

All data collected and analyzed within this study are available from the corresponding author on reasonable request.
