# Supplementary material for: The secretor status of blood group antigens in the saliva in people with oral cancers: a systematic review
Source: Syst Rev. 2024 Jan 2;13:13. doi: 10.1186/s13643-023-02399-8 (PMC10763318; doi:10.1186/s13643-023-02399-8)
Supplement: Supplementary file 1 — Additional file 1. [file 13643_2023_2399_MOESM1_ESM.pdf]

Number #	Searches	Results
1	ABO BloodGroup System/ or ABO blood group*.mp.	16612
2	ABO blood group antigen*.mp.	215
3	"Blood Grouping and Crossmatching"/	4847
4	ABO blood group type*.mp.	27
5	ABO blood type*.mp.	653
6	ABO.mp.	20972
7	blood type*.mp.	3305
8	Blood group*.mp. or Blood Group Antigens/	52003
9	1 or 2 or 3 or 4 or 5 or 6 or 7 or 8	55846
10	Saliva/ or Saliva*.mp.	130260
11	salivation.mp. or Salivation/	6306
12	saliva analysis.mp.	133
13	saliva.tw.	50106
14	10 or 11 or 12 or 13	130260
15	Mouth/	23487
16	Lip/	11810
17	Gingiva/	18919
18	Tongue/	19662
19	Oropharynx/	4689
20	Hypopharynx/	2177
21	Palate/	10784
22	Cheek/	7860
23	15 or 16 or 17 or 18 or 19 or 20 or 21 or 22	92681
24	(Mouth/ or Lip/ or Gingiva/ or Tongue/ or Oropharynx/ or Hypopharynx/ or Palate/ or Cheek/) adj3 (cancer* or neoplasm* or tumor* or malignancy* or carcinoma* or dysplasia*).mp. [mp=title, abstract, original title, name of substance word, subject heading word, floating sub-heading word, keyword heading word, organism supplementary concept word, protocol supplementary concept word, rare disease supplementary concept word, unique identifier, synonyms]	14088

25	Mouth Neoplasms/ or Mouth Neoplasm*.mp	38377
26	Lip Neoplasms/	4313
27	Gingival Neoplasms/	2432
28	Tongue Neoplasms/	10629
29	Oropharyngeal Neoplasms/	6765
30	Hypopharyngeal Neoplasms/	3405
31	Palatal Neoplasms/	3031
32	(oral adj5 (cancer* or neoplasm* or tumor* or malignancy* or carcinoma or dysplasia*)).mp. [mp=title, abstract, original title, name of substance word, subject heading word, floating sub-heading word, keyword heading word, organism supplementary concept word, protocol supplementary concept word, rare disease supplementary concept word, unique identifier, synonyms]	44155
33	(Lip Neoplasm* or Gingival Neoplasm* or Tongue Neoplasm* or Oropharyngeal Neoplasm* or Hypopharyngeal Neoplasm* or Palatal Neoplasm*).mp.	28629
34	24 or 25 or 26 or 27 or 28 or 29 or 30 or 31 or 32 or 33	88650
35	9 and 14 and 34	13
